# Oxygen: The Rate-Limiting Factor for Episodic Memory Performance, Even in Healthy Young Individuals

**DOI:** 10.3390/biom10091328

**Published:** 2020-09-17

**Authors:** Gil Suzin, Tom Halpert Frolinger, Dror Yogev, Amir Hadanny, Merav Catalogna, Yuri Rassovsky, Shai Efrati

**Affiliations:** 1 The Sagol Center for Hyperbaric Medicine and Research, Shamir (Assaf-Harofeh) Medical Center, Zerifin 70300, Israel; halpertom@gmail.com (T.H.F.); dror_yogev@yahoo.com (D.Y.); amir.had@gmail.com (A.H.); merav@catalogna.co.il (M.C.); efratishai@outlook.com (S.E.); 2 Department of Psychology, Bar Ilan University, Ramat-Gan 5290002, Israel; yurir@biu.ac.il; 3 Sackler School of Medicine, Tel-Aviv University, Tel-Aviv 6997801, Israel; 4 Leslie and Susan Gonda (Goldschmied) Multidisciplinary Brain Research Center, Bar-Ilan University, Ramat-Gan 5290002, Israel; 5 Department of Psychiatry and Biobehavioral Sciences, UCLA Semel Institute for Neuroscience and Human Behavior, Los Angeles, CA 90095, USA; 6 Sagol School of Neuroscience, Tel-Aviv University, Tel-Aviv 6997801, Israel

**Keywords:** episodic memory, hyperoxia, hyperbaric oxygen, cognitive enhancement

## Abstract

Cognition is a crucial element of human functionality. Like any other physical capability, cognition is both enabled and limited by tissue biology. The aim of this study was to investigate whether oxygen is a rate-limiting factor for any of the main cognitive domains in healthy young individuals. Fifty-six subjects were randomly assigned to either increased oxygen supply using hyperbaric oxygen (two atmospheres of 100% oxygen) or to a “sham” treatment (a simulation of increased pressure in a chamber with normal air). While in the chamber, participants went through a battery of tests evaluating the major cognitive domains including information processing speed, episodic memory, working memory, cognitive flexibility, and attention. The results demonstrated that from all evaluated cognitive domains, a statistically significant improvement was found in the episodic memory of the hyper-oxygenized group. The hyper-oxygenized group demonstrated a better learning curve and a higher resilience to interference. To conclude, oxygen delivery is a rate-limiting factor for memory function even in healthy young individuals under normal conditions. Understanding the biological limitations of our cognitive functions is important for future development of interventional tools that can be used in daily clinical practice.

## 1. Introduction

Cognition, “the mental action or process of acquiring knowledge and understanding through thought, experience, and the senses,” [1] is crucial for human functionality. Cognition is the sum of different aspects of intellectual domains such as attention, memory and working memory, processing speed, cognitive flexibility, and executive functions. Similar to any other physical capability, cognition is both enabled and limited by tissue biology, in this case, brain biology. Most research on the biology of cognition relates to pathophysiological conditions and how they cause cognitive decline, and less is known about the biological rate-limiting factors that prevent us from enhancing cognitive functions. In this study, we challenged the different cognitive domains to evaluate whether, in normal healthy brains, oxygen delivery is a rate-limiting factor preventing enhanced cognitive performance.

The brain has unique thermodynamic characteristics. It comprises about 2% of the body’s total weight, yet it utilizes about 20% of the total oxygen supply and consumes about 30% of the body’s total energy. At normal oxygen (normoxic) conditions, oxygen is continuously consumed by the brain tissue, and brain tissue oxygenation (PbTO2) values range from a maximal intra-capillary 90 mmHg to less than 30 mmHg [2]. At any given moment, the brain utilizes all oxygen delivered, and the perfusion differentially changes based on neuronal activity, as demonstrated and utilized by functional MRI. When neurons become active, local blood flow to those brain regions increases at the expense of other less active brain regions [3]. Apparently, since oxygen is a limited resource, many neurological functions are regularly activated at suboptimal levels. Therefore, it is safe to assume that cognition is another such affected function. 

Data relating to the dependency of different cognitive functions on brain oxygenation have been mostly gathered from pathologic conditions [4,5]. Previous research on hypoxia has demonstrated a decline in cognitive function when oxygen’s partial pressure goes below 50 mmHg [6]. In such cases, there is a decline in memory performance [7,8], attention skills [9], working memory [10], and executive functions [11]. 

Very few studies have investigated data on the immediate effects of increasing oxygen delivery to the brain (hyperoxia) on cognitive function. Scholey et al. demonstrated that a short period of hyperoxia, induced up to five minutes prior to learning a set of words, can enhance later word recall [12]. Chung et al. have demonstrated that doubling the breathing oxygen concentration to 40% oxygen administration leads to increases in the N-back task performance [13]. In addition, in a previous study, we demonstrated that oxygen is indeed a rate-limiting factor for performing a multitask paradigm (motor + cognitive tasks) [14]. 

The aim of the current study was to investigate whether significant hyperoxia can enhance major cognitive domains in healthy young people.

## 2. Materials and Methods 

The study was a prospective, randomized, sham-controlled trial, conducted at the Sagol Center for Hyperbaric Medicine and Research at the Shamir Medical Center, Israel. The protocol was approved by the Shamir Institutional Review Board (0031-17-ASF) and was registered in the US National Institute of Health Clinical Trails, registry number NCT04358796. All participants signed an informed consent form prior to their inclusion. 

### 2.1. Participants

The study included healthy volunteers, between the ages of 20 and 39 years old. Candidates were recruited via notification in social media (i.e., Facebook groups for students). Candidates were excluded if they had any chronic disease, any history of brain insult such as traumatic brain injury, stroke, encephalitis or meningitis, any change in their cognitive or behavioral functions in the month prior to their inclusion, any chest pathology or lung disease incompatible with pressure changes, any ear or sinus pathologies incompatible with pressure changes, if they were treated by hyperbaric oxygen therapy (HBOT) for any reason prior to study enrollment, or if they were not able to read and sign the informed consent form.

### 2.2. Protocol

After signing the informed consent form, participants were randomized, according to a randomization table, to perform the cognitive tasks in one of two environments within a multiplace hyperbaric chamber (HAUX-STARMED 2700/5): (a) HBOT group—the chamber environment was compressed to 2 absolute atmospheres (ATAs), and participants breathed 100% oxygen by personal mask while doing the cognitive tests; (b) sham group—participants breathed 21% oxygen at 1 ATA (normal air). To simulate the pressure sensation, the chamber was compressed to 1.1 ATA during the first five minutes of the session with the noise of circulating air, and then decompressed gradually during the next 15 min to 1.0 ATA. In the last five minutes of the session, circulating air noise was used to simulate decompression. In order to avoid the training effect, the paradigm was administered only once without crossover between the interventions. 

#### 2.2.1. Training Session

Prior to the evaluation session, to minimize learning effects and familiarize the participants with the trial’s cognitive tasks, all participants attended a training session. All task instructions were reviewed and participants practiced them in a normal environment of 1 ATA, breathing room air. 

#### 2.2.2. In-Chamber Evaluation Procedure

Both groups were tested following compression/sham compression and after 20 min of wearing a mask to allow adjustment to the environment. Tests were administered simultaneously to the two groups, in two separated chambers, using the same audio-recorded instructions. Subjects performed the tasks using paper and pencil. Tests were scored by two blinded research assistants (T.H.F. and D.Y.).

### 2.3. Outcome Measures

**Figure 1:**
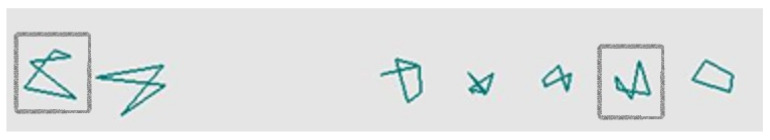
A trial from the cognitive processing speed test (gray frames were added for illustration purposes).

**Figure 2:**
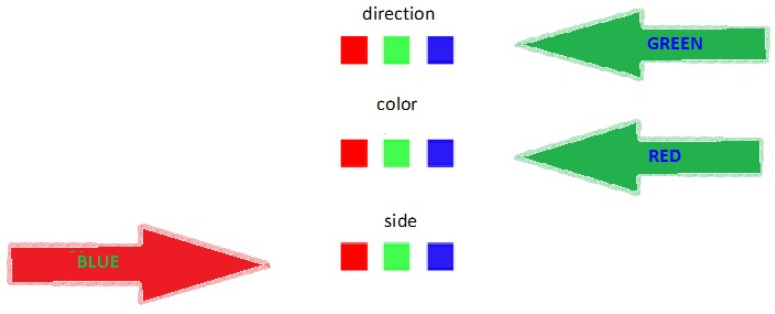
An example of three trials from the augmented Stroop task. The three colored boxes in the center of each trial were used to indicate either the location of the correct answer (left/right) or the color of the right answer (red/green/blue). The correct sequence in the three trials above would be “red—blue—red.”

**Figure 3:**
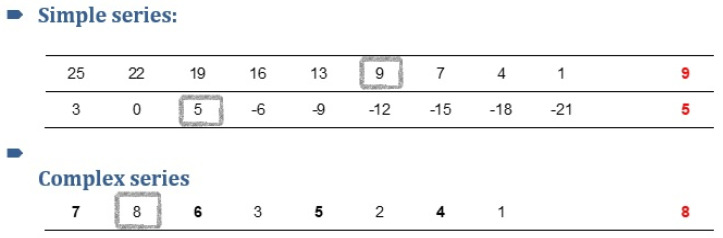
An example of two simple series and one complex series. Grey frames and the answers in red on the right side were added for illustration purposes.

Cognitive function was evaluated using a battery comprising eight tests producing objective measures of accuracy and response time (see Table 1). The battery was designed to fit a highly functioning group of participants wearing masks. The evaluated cognitive domains included the following:

- Working memory:
  ○ Auditory N-back test—In this test, the participants listened to a sequence of stimuli and were required to state whenever the presented stimulus matched the one preceding “n” steps earlier in the sequence. In the classic auditory N-back test, the participant hears a sequence that consist of letters. The N-back task captures the active part of working memory when *N* ≥ 2 [15]. In the current paradigm, we played a recording of six 20-digit strings read out loud at a constant pace. In the first stage, the instructions were to note whenever there was *N* = 1 repetition (i.e., “1”—“1”). In the second stage, the instructions were to note whenever there was *N* = 2 repetition (i.e., “1”—“3”—“1”). The accuracy score reflected the percentage of correctly traced repetitions out of the total repetitions from the second stage sequences. The number sequences were randomized using MATLAB version 7.10.0 (R2010a, The MathWorks Inc., Natick, MA, USA), while maintaining a rule that in each sequence there will be valid repetitions at a 1:3 probability.
  ○ Digit-span test—In this test, a list of random numbers was read out loud at the rate of one per second. Subjects were asked to write as many digits as they recalled from each string. There were two versions of this test. In the forward version, the subjects recalled the sequence in the order that it was read. In the backward version, the subjects recalled the sequence in reverse order (i.e., from the last to first number read). Standard administration of the test started with a three-digit sequence and ended after the subject made two consecutive errors [16]. In the current research, we opted to prevent ceiling effects. Therefore, the initial sequence included seven digits in the forward version and five digits in the backward version. The test followed a standard procession in which there were two trials for each sequence length, and the subjects were required to recall them all until the test ended at sequence length = 10 in the forward test and sequence length = 8 in the backward test. Two scores were given for each version of the test. The “span score” is the highest sequence length correctly recalled. The “accuracy score” is the sum of digits correctly recalled in their position, across the whole test, regardless of the accuracy of the full sequences. To construct this test, random number sequences were generated for each sequence length.
- Divided attention: In this test, the subjects were instructed to divide attention between a visual search task (i.e., searching for the cluster 1-7-4 and circling all of its occurrences, among a long string of randomized numbers) and an auditory tracing task (i.e., recognizing verbally presented words containing the Hebrew letter “מ” (mem), which is equivalent to the English letter “M”, and writing them down). The words in the test were sampled from dictation lists for children in the third grade. Words containing the letter “מ” appeared among other words in random intervals. The test ended when all of the words were verbally presented (using a prepared recording). The score is the percent of correct responses, summed up from both channels.
- Episodic memory: In this test, subjects were required to pass a version of the California verbal learning test (CVLT) [17], which is a widely used episodic memory test. The test procedure consisted of two parts: (1) immediate learning—hearing a list of 16 nouns that are drawn from four semantic categories (i.e., fruits, clothes, etc.) and recalling as many words as possible immediately afterward. The order of words in the list was randomized. The list was read for a second time (after re-randomization of words). The score for this part is the sum of words retrieved in both trials. (2) Interference—recalling words from a second list of 16 nouns and then, once again, retrieving words from the initial list (without another exposure). The score for this part is total number of words recalled from the first list after interference.
- Cognitive processing speed: In this test, subjects solved a symbol search paradigm known to be a valid measure of cognitive processing speed in healthy older adults [18]. The assignment was time-limited and lasted five minutes. For each trial, two target symbols were presented on the left side of the paper. The subjects had to decide whether one of the two targets appeared in a series of five shapes on the right (shapes could reappear on the right in different sizes and/or different orientations) (see Figure 1). The score for this test is the sum of the correct responses.
- Cognitive flexibility: In this test, subjects had to solve an augmented Stroop-like paradigm. Stroop tasks require subjects to inhibit their response to an attribute of the stimuli to attend to a competing attribute of the stimuli. The traditional Stroop-test requires the subject to neglect the content attribute of a word stimulus (e.g., “green”) to attend to the color attribute of the word (e.g., red font). The Stroop test is known to measure “response inhibition” [19]. In the current version of the test, subjects were shown colored arrows, either on the left or the right side of the page, either pointing to the left or to the right, with a “color name” written in the middle. The “color name” font color contradicted the color of the arrow (see Figure 2). The subjects had to attend either to the side of the page the arrow was on (“side”), to the direction the arrow pointed (“direction”), or to the color of the font of the word written on the arrow (“color”). The instructions changed intermittently between trials, and the test was timed (five minutes). The target score was the sum of correct responses.
- Executive function (problem-solving): In this test, participants were required to solve an arithmetic series and trace irregular elements embedded in them, a task that is known to reflect executive functions (problem-solving) [20]. In the simple series part, subjects were given three minutes to solve up to 27 series. In the complex series part, they were given three minutes to solve up to 27 complex series, each composed of two interlocked series (see Figure 3). The score comprised the number of correct responses in each part.

### 2.4. Statistical Analyses

The data were analyzed using SPSS v.27. The results of the HBOT group were compared to those of the sham group using an independent *t*-test (for two samples). An FDR (False Discovery Rate) correction for multiple comparisons was performed. The effect size was calculated using Cohen’s D. The statistical significance was defined as *p* < 0.05 for all statistical analyses. 

## 3. Results

**Figure 4:**
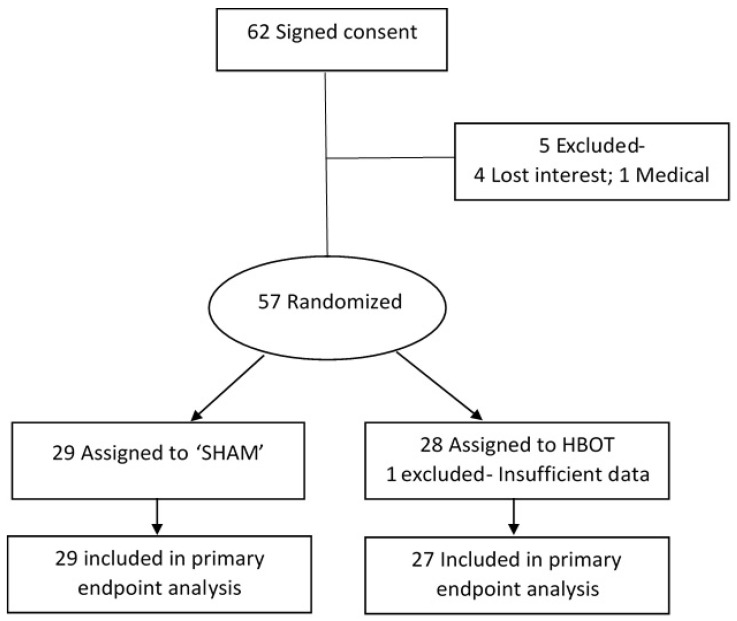
Subject inclusion flow diagram.

Sixty-two subjects signed the informed consent form and were randomized to either study group. Out of the 62, 5 did not perform an in-chamber test and were excluded: 1 had intercurrent disease that prevented him from going into the chamber and 4 lost interest after the training session. Another subject did not complete the tests in the chamber. Accordingly, 56 individuals were included in the final analyses, 27 subjects in the HBOT group and 29 in the sham group (see Figure 4).

The baseline characteristics are presented in Table 2.

The mean age of the participants was 27.42 ± 4.35; 51.7% were female and the average number of years of education among them was 14.98 ± 1.92. There was no significant difference in any of the participants’ characteristics between the two groups (see Table 2). The cognitive test results are summarized in Table 3. The learning curve, as demonstrated by the total number of words recalled from a list of words read repetitively, significantly improved while breathing hyperbaric oxygen in comparison to subjects from the sham group. Subjects from the HBOT group recalled significantly more words in total compared to the sham group (*t* = 4.76, *p* < 0.03), although this result did not withstand the FDR correction. The most notable difference between the groups was in memory decay. Participants in the HBOT group were less vulnerable to memory decay, as indicated by their preferable retrieval rates after distraction (i.e., a trial in which they had to memorize and recall a new set of words) (*t* = 15.1, *p* < 0.001). Cohen’s D revealed a strong effect size (0.85) indicating that the significant difference between the experimental groups was also clinically valuable. There was no other significant difference in any of the other evaluated cognitive parameters (see Table 3).

## 4. Discussion

In this study we evaluated whether oxygen delivery serves as a rate limiting factor for any of the major cognitive domains. Fifty-six young healthy subjects were randomized to an increased-oxygenation condition in a hyperbaric oxygen chamber or to a “sham” chamber, where they were administered a battery of cognitive tests. From all the evaluated cognitive domains, we found that episodic memory was significantly enhanced by increased oxygen delivery. The learning curve, as demonstrated by the total number of words recalled from a list of words read repetitively, was significantly improved while breathing hyperbaric oxygen compared to subjects from the sham conditions. Subjects in the hyper-oxygenated group were also able to preserve more words following an interference. None of the other measured cognitive domains was enhanced by increased oxygen delivery.

Episodic memory, the human capacity to remember past events (i.e., places, figures, discussions, emotions, etc.), is essential for an experience of continuity that is foundational to personal identity. In a sense, “fine-grained” episodic memory is one of the hallmarks of humanity. Episodic memory involves a complex network encompassing multiple brain regions that are highly dependent on oxygen delivery (i.e., the PFC (Pre Frontal Cortex) and the parietal and medial temporal lobe (MTL) regions) [21]. In its essence, the episodic memory circuit requires higher-association areas to process the sensory information (the neocortex), interface areas to communicate with the hippocampus (the parahippocampal region), the hippocampus to integrate and retrieve information about the episode, and executive areas to produce the appropriate behavior (the prefrontal cortex) [22]. Specifically, the hippocampus, with its related structures in the medial temporal lobe (MTL), is crucial for episodic memory [23]. Inputs from the entorhinal cortex and the perirhinal cortex indicate that complex object relations are integrated in the hippocampus, where their representations are preserved [24]. Any decline in oxygen delivery has significant effects on the hippocampus and memory function [25,26,27]. Accordingly, episodic memory is not a steady trait and is very sensitive to environmental and/or physiological changes [28,29]. Episodic memory is the most age-sensitive system, with an average onset of decline around 60 years of age [23,30]. Notably, the results of this study indicate that memory function is a continuum that does not reach its maximal ceiling effect at the normal sea-level environment, even in healthy young individuals. 

Episodic memory, along with semantic memory, comprises the category of explicit memory, one of the two major divisions of long-term memory (the other being implicit memory). The understanding that oxygen is a rate-limiting factor for episodic memory even in healthy young adults and the insight that memory can be further enhanced by being in a hyper-oxygenized environment may explain why repressed memories can be recovered during hyperbaric oxygen therapy (HBOT). There is cumulative evidence that patients coming to HBOT recover long-term repressed traumatic memories, and in some of the hyperbaric centers, recovery of repressed memory was added as a potential side effect to the informed consent form [31]. Surfacing of inaccessible memories can be crucial for treating diseases like PTSD and other amnestic conditions where knowing the history is essential. Understanding the biology responsible for our ability to recall specific experiences and understanding that this ability can be enhanced by HBOT gives us a powerful tool that can be used for those who need it.

The finding that not all cognitive measures are improved by oxygen administration is in agreement with results from other studies. For example, the level of working memory on a computerized test was unaffected by oxygen administration [12,32]. Hyperoxia had no effect on forward or backward digit span performance [12]. The main potential claim against those previous studies could be that the oxygen delivery was not increased enough to conclude that oxygen is not a limiting factor for the other cognitive functions. However, in the current study, oxygen delivery was significantly increased by the use of hyperbaric oxygen compressed to 2 ATAs while breathing 100% oxygen (more than a 15-fold increase in partial pressure of blood oxygenation), and these results hold. This means that oxygen is not the limiting factor for all cognitive domains.

This study had several limitations that should be noted. First, the relatively homogenous sample comprised highly educated subjects with high performance could make it harder to detect significant differences. It is possible that inclusion of a more diverse set of subjects (i.e., diverse levels of education and/or different cultural backgrounds) would lead to different results. Second, the cognitive tests included several adjustments needed for a chamber setting. It is important to note though that the cores of all versions were based on well-validated paradigms. The small adjustments were made to fit a cohort of healthy and high-functioning individuals having the tests done in a group context, inside an oxygen chamber (i.e., with masks on their faces). Third, even though the protocol included a significant increase in oxygen delivery by the use of a hyperbaric chamber, it is still possible that a longer duration of exposure or higher oxygen pressure would result in different findings. Fourth, although the results demonstrate an elevation in memory performance, it is not clear whether these are due to a better encoding of the verbal information or a better process of consolidation. Further studies and other paradigms (i.e., [33]) should be used to figure this out.

## 5. Conclusions

Oxygen delivery is a rate-limiting factor for episodic memory even in healthy young individuals at normal sea-level conditions. Understanding the biology behind cognitive functions and the ability to enhance them may serve as an interventional tool that can be used in daily clinical practice.

## Figures and Tables

**Table 1:** Domains and measures used in the study.

Domain	Test	Primary Outcome Measure
Working memory	N-back	Percentage accuracy
	Digit span	Span score; accuracy score
Divided attention	Multi-task	Percentage accuracy
Episodic memory	Word lists	Sum immediate retrieval; retrieval after interference
Cognitive processing speed	Symbol search	Sum accuracy
Cognitive flexibility	Stroop	Sum accuracy
Problem solving	Arithmetic	Sum accuracy

**Table 2:** Baseline characteristics.

	HBOT (N = 27)	SHAM (N = 29)	*p* Value
**Mean Age (Stdev)**	27.92 ± 4.77	26.96 ± 3.95	0.41
**Years of Education (Stdev)**	15.36 ± 2.11	14.64 ± 1.7	0.178
**Gender (% Females)**	59%	45%	0.28

Differences between groups in “Mean Age” and “Years of Education” were calculated using an unpaired *t*-test. Differences between groups in “% females” were calculated using the chi-square test.

**Table 3:** Cognitive domains results.

Cognitive Domain	HBOT (N = 27)	SHAM (N = 29)	*t*-Test (*p*)	FDR (*p*)	Effect Size (Cohen’s D)
Digit span (forward: highest correct)	7.78 (1.5)	7.46 (0.85)	0.37	0.55	0.26
Digit span (forward: overall correct)	41.3 (11.98)	38.27 (6.55)	0.25	0.75	0.31
Digit span (backward: highest correct)	7.34 (1.52)	6.85 (1.81)	0.28	0.67	0.29
Digit span (backward: overall correct)	40.85 (10.62)	42 (9.04)	0.66	0.79	0.12
Stroop (% correct)	59.03 (15.39)	62.96 (15.99)	0.35	0.6	0.25
Multi-tasking (% total accuracy)	66.6 (7.97)	64.24 (8.47)	0.29	0.58	0.29
Symbol search (no. correct)	31.66 (6.3)	33.03 (8.47)	0.49	0.65	0.18
N-back (% correct)	91.19 (9.57)	90.42 (10.05)	0.76	0.83	0.08
Series simple (no. correct)	15.3 (5.9)	15.27 (5.48)	0.98	0.98	0.00
Series complex (no. correct)	12 (4.51)	13.41 (4.35)	0.23	0.92	0.32
CVLT (total correct)	22.4 (2.96)	20.51 (3.75)	**0.04**	0.24	0.54
CVLT (no. of words—post interference)	11.33 (2.3)	9.03 (2.93)	**0.001**	**0.012** *	0.85

Bold—*p* < 0.05; *—Satisfies FDR (False Discovery Rate) corrections. (Stdev in parentheses).
